# Restricted loss of olivocochlear but not vestibular efferent neurons in the senescent gerbil (*Meriones unguiculatus*)

**DOI:** 10.3389/fnagi.2015.00004

**Published:** 2015-02-13

**Authors:** Susanne Radtke-Schuller, Sabine Seeler, Benedikt Grothe

**Affiliations:** ^1^Division of Neurobiology, Department Biology II, Ludwig-Maximilians-UniversityMunich, Germany; ^2^IFB German Center for Vertigo and Balance DisordersMunich, Germany

**Keywords:** aging, cholinergic efferent systems, brainstem, olivocochlear neurons, superior olivary complex, vestibular, trigeminal, auditory

## Abstract

Degeneration of hearing and vertigo are symptoms of age-related auditory and vestibular disorders reflecting multifactorial changes in the peripheral and central nervous system whose interplay remains largely unknown. Originating bilaterally in the brain stem, vestibular and auditory efferent cholinergic projections exert feedback control on the peripheral sensory organs, and modulate sensory processing. We studied age-related changes in the auditory and vestibular efferent systems by evaluating number of cholinergic efferent neurons in young adult and aged gerbils, and in cholinergic trigeminal neurons serving as a control for efferents not related to the inner ear. We observed a significant loss of olivocochlear (OC) neurons in aged compared to young adult animals, whereas the overall number of lateral superior olive (LSO) cells was not reduced in aging. Although the loss of lateral and medial olivocochlear (MOC) neurons was uniform and equal on both sides of the brain, there were frequency-related differences within the lateral olivocochlear (LOC) neurons, where the decline was larger in the medial limb of the superior olivary nucleus (high frequency representation) than in the lateral limb (middle-to-low frequency representation). In contrast, neither the number of vestibular efferent neurons, nor the population of motor trigeminal neurons were significantly reduced in the aged animals. These observations suggest differential effects of aging on the respective cholinergic efferent brainstem systems.

## Introduction

Hearing deficits (presbycusis) and vertigo are symptoms of age-related auditory and vestibular disorders reflecting multifactorial changes in the peripheral and central nervous system. Presbycusis is also characterized by reduced speech recognition especially in noisy environments, slowed central processing of acoustic information, and impaired sound localization. The auditory and vestibular systems both evolved from the octavolateralis system (for review, see Köppl, 2011). Both sensory modalities use the same type of sensory receptor cells, i.e., hair cells, which receive direct efferent innervation by neurons of the same ontogenetic descent from rhombomere 4 (Bruce et al., 1997; Simmons, 2002). This cholinergic efferent synaptic transmission is a unique feature among sensory systems (for review, see Roberts and Meredith, 1992). Originating bilaterally in the brain stem, auditory efferent neurons and vestibular efferent neurons are the last links in elaborate descending neural pathways of the central auditory and vestibular systems (for reviews, see Holt et al., 2011; Schofield, 2011). These cholinergic descending projections exert central feedback control on the peripheral sensory organs, thereby modulating afferent information processing.

In mammals, the efferent olivocochlear (OC) system originates in the superior olivary complex (SOC) and consists of different components. Large medial olivocochlear (MOC) neurons are mainly located in the medial periolivary region (mainly in the ventral nucleus of the trapezoid body (VNTB). Additionally, some large OC neurons in the dorsal periolivary nucleus (DPO) are described as a sub-group of MOC neurons (rodents: Aschoff and Ostwald, 1987; Brown and Levine, 2008; cat: Warr et al., 2002). Small lateral olivocochlear (LOC) neurons are situated in and around the lateral superior olive (LSO) and display a discrete tonotopic projection to the cochlea (Guinan et al., 1984; Robertson et al., 1987). An additional group of larger LOC neurons, so-called shell neurons, is found at the margins of the LSO (Vetter and Mugnagnaini, 1992) and projects also tonotopically but to a broader frequency range (Warr et al., 1997). This sub-group is not as distinct in the gerbil as in other rodents and is therefore subsumed in the group of LOC neurons in this study. There can be considerable variation in the location, number and ratio of LOC and MOC neurons in gerbils (Aschoff et al., 1988; Kaiser et al., 2011). All OC neurons operate with acetylcholine as synaptic transmitter, and LOC neurons in addition use a variety of co-transmitters (Sewell, 2011).

Functionally, MOC neurons modulate the electromechanical amplification and gain in outer hair cells and thereby adjust the auditory nerve’s dynamic range. They contribute to the protection of the auditory system from acoustic trauma and contribute to extracting biologically important acoustic signals in noise (Guinan, 2011). Age-related decline of MOC functionality prior to outer hair cell degeneration has been demonstrated with distortion product otoacoustic emission (DPOAE) measurements in humans (Kim et al., 2002) and in CBA mice (Jacobson et al., 2003).

The function of the LOC system is less understood, but its neurons are known to modulate afferent activity of type I auditory nerve fibers through various neurotransmitter systems. The LOC efferents potentially protect the ear from acoustic overexposure and/or may balance the sensitivity of the two ears (Ruel et al., 2001; Darrow et al., 2006). Other reports have recently shown that the efferent auditory feedback neurons may have a protective function that slows down the progression of age-related cochlear hearing loss (Liberman et al., 2014). Age-related synaptic loss of MOC terminals and changes in LOC efferent innervation pattern has been shown to occur prior to hearing loss (Fu et al., 2010; Lauer et al., 2012), but it is still unknown whether age-related synaptic change is the cause or the consequence of neuronal cell loss (Jin et al., 2011). However, the age-related loss of OC neurons has not yet been evaluated directly.

The small group of efferent vestibular neurons is located in the medullary brainstem and similarly organized throughout mammals (gerbil: Perachio and Kevetter, 1989; Purcell and Perachio, 1997; Holt et al., 2011). About 90% of the efferent neurons belong to the larger group called “group *e”* (Goldberg and Fernandéz, 1980) and are located dorsolateral to the facial nerve genu between the abducens and superior vestibular nuclei. These neurons are anti-cholinetransferase (ChAT) immunopositive and in addition use a variety of co-transmitters (Perachio and Kevetter, 1989; Ryan et al., 1991), whereas neurons of the smaller group ventral to the genu do not stain for any of these markers (Perachio and Kevetter, 1989) and are not discussed further in this study. The responses of efferents and their effect on afferent discharge have been studied in detail (Holt et al., 2011), but the functions of the efferent vestibular system still remain elusive.

The objective of this study was to quantify and compare age-related loss of cholinergic efferent auditory and vestibular feedback neurons in senescent gerbils, a model species for human hearing (Ryan, 1976; Cheal, 1986). This decline was compared with age-related cell loss in the cholinergic motor trigeminal system. The observations suggest a differential impact of aging on these cholinergic brainstem systems.

## Materials and methods

### Animals

Immunolabeling was conducted on the brain stem of 10 healthy Mongolian gerbils (*Meriones unguiculatus*) of both genders. The ages of the animals were either 3–5 month (young adults, *n* = 5) or 2.5–3.5 years (aged animals, *n* = 5). Animals were provided by the breeding facility of the Biocenter of the University of Munich (LMU). All experiments followed regulations on animal welfare approved by the Bavarian state government (AZ. Reg. v. Obb. 55.2-1-54.2531.8-211-10) and the European Communities Council Directive (86/609/EEC).

### Tissue processing

**Figure 1:**
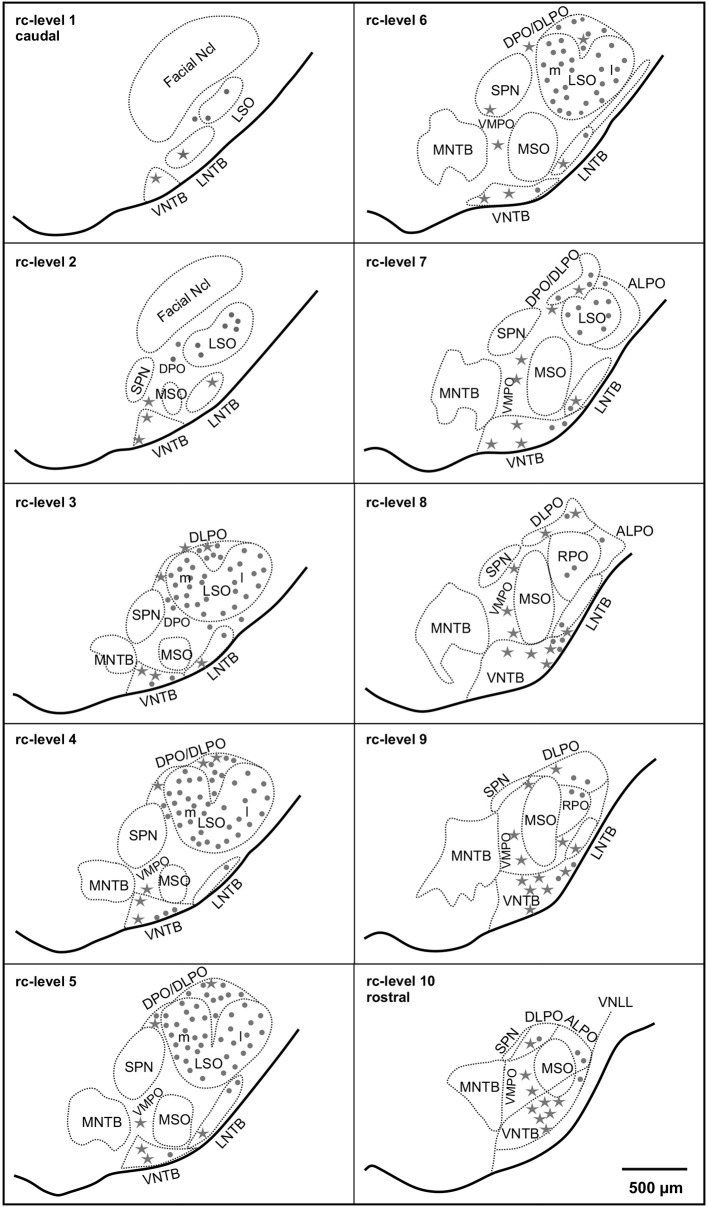
**Distribution of cholinergic neurons in the superior olivary complex (SOC) of the gerbil (*M. unguiculatus*)**. Reference series shows ventral outlines of the brainstem (solid lines) and the subdivisions of the SOC (dotted contours) at 10 rostrocaudal (rc) positions from facial nucleus (Facial Ncl) to the ventral nucleus of the lateral lemniscus (VNLL). Rc-level 1 indicates the most caudal and rc-level 10 the most rostral position. Dots: small cholinergic neurons comprising LOC neurons. Stars: large cholinergic neurons, including MOC neurons. The density of symbols represents roughly the relative frequency of occurrence of the respective cholinergic neurons within each subdivision.

Gerbils were anesthetized by a lethal dose of i.p. administered Narcoren® (Merial GmbH, Halbergmoos, Germany) (200 mg/kg body weight). After the animals had reached a deep anesthetic state marked by a complete loss of the flexor reflex at all limbs, they were perfusion-fixed, first with Ringer solution supplemented with 0.1% heparin (Meditech Vertriebs GmbH, Parchim, Germany) and then with 4% paraformaldehyde (PFA). Brains were post-fixed in 4% PFA solution for at least 2.5 h up to overnight. Subsequently, the brains were oriented along standardized coordinates in an embedding chamber that was then filled with agarose. The standardized alignment before embedding guaranteed that the brains were cut in planes that corresponded as closely as possible to each other inter-individually and to a reference series (Figure 1). The resulting blocks were trimmed and the regions of interest were cut with a Leica VT 1200S vibratome (Leica Biosystems, Nussloch Germany) into 40 µm thick coronal sections of the brainstem. In order to achieve optimum comparability of results in young adult and aged animals, all procedures for tissue preparation and immunolabeling were applied to pairs or groups of young adult and aged healthy individuals together.

### Immunolabeling

We used ChAT antibody as marker for cholinergic neurons (Hedreen et al., 1983; Kaiser et al., 2011). Anti-chondroitinsulfate (CSPG) antibody was applied as a marker for perineuronal nets. Perineural nets are chondroitin sulfate proteoglycans which form an extracellular matrix that surrounds many neuronal somata, dendrites and synapses in net-like structures all over the brain. They ensheath most SOC principal neurons (gerbil: Lurie et al., 1997), and the neurons of the brainstem motor nuclei (including the motor trigeminal nucleus). CSPG antibody was therefore well-suited as an overview stain to delineate the SOC nuclei. Anti-microtubule-associated protein 2 (Map2) antibody was used to stain neuronal somata and dendrites.

The sections were washed and non-specific binding sites were saturated with a blocking solution containing 1% BSA, 1% Triton X-100 and 0.1% saponin for 1 h, then incubated in the primary antibody mix (diluted in blocking solution) at 4°C on a shaker. To ascertain the best incubation time, test sections were either incubated overnight, or incubated for two nights. The prolonged incubation time resulted in more intense staining but did not lead to an increased number of immunoreactive cells or a higher background. The specificity of all primary antibodies used has been previously published and the relevant publications are indicated for the respective antibodies. The primary antibodies used were: goat anti-ChAT (1:500 Millipore, AB144P; Kaiser et al., 2011), mouse anti-CSPG (1:500 Millipore, MAB5284; Andrews et al., 2012), chicken anti-Map2 (1:1000, Neuromics, CH22103; e.g., Rautenberg et al., 2009). Following extensive washing the sections were incubated with a combination of fluorescent secondary antibodies of different wavelength in blocking solution (1:400) for 4 h at room temperature or 18 h at 4°C on the shaker in the dark (donkey anti goat (Alexa Fluor 488 Dianova 705-546-147), donkey anti mouse (Cy3, 570 nm; Dianova 715-166-151) and donkey anti chicken (Alexa Fluor 647 Dianova 703-606-155). After exhaustive washing, the sections were mounted with Vectashield® (Vector, Burlingame, CA, USA) and sealed with nail polish.

### Image acquisition

**Figure 2:**
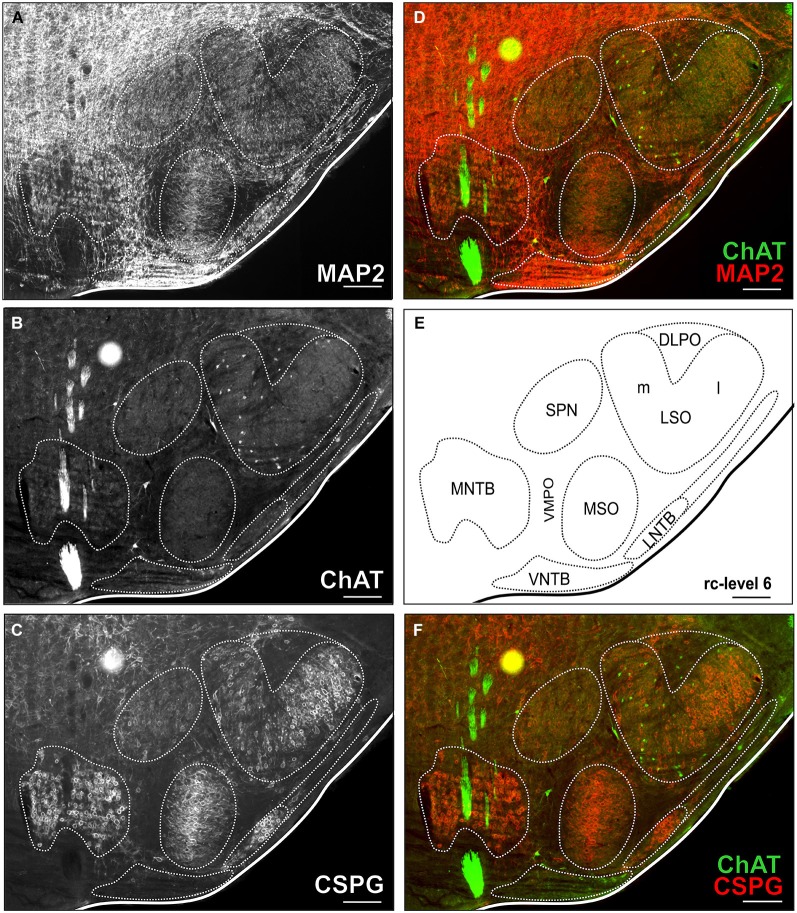
**Triple-stained example section of the SOC region of a young adult gerbil**. The section corresponds to the reference series at rc-level 6 **(E)**. In **(A,B,C)** MAP2 stain, ChAT stain and CSPG stain are depicted alone with delineated SOC nuclei. Overlays of ChAT (Alexa Fluor 488, green) and MAP2 (Alexa Fluor 647, red) are shown in **(D)** and ChAT and CSPG (Cy 3, 570; red) in **(F)**. ChAT positive cells were easily detected in the ChAT stain alone as well as in overlay with both overview stains (MAP and CSPG). Scale bar in **(A–F)**: 500 µm.

For delineation of SOC nuclei and counting of cholinergic neurons, sections were imaged with a virtual slide microscope (VS120 S1, Olympus BX61VST, Olympus-Deutschland, Hamburg, Germany) at 10× magnification using the proprietary software dotSlide® (Olympus). All three colors of the secondary antibodies used for immunostaining were acquired sequentially and could be visualized separately or in overlay (Figure 2).

**Figure 3:**
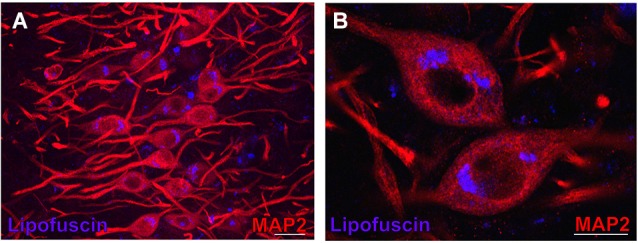
**Lipofuscin granules in medial superior olive (MSO) neurons of an aged gerbil**. MSO neurons are MAP2 immunostained (Alexa Fluor 647, red). Lipofuscin granules have been excited with the DAPI excitation wavelengths and appear blue. Confocal images show a maximum projection of image stacks in **(A)** and a single optical image of 0.3 µm thickness in the enlargement in **(B)**. Scale bar in **(A)**: 50 µm and 20 µm in **(B)**.

For counts of total neuron numbers in LSO, confocal optical sections were acquired with a Leica TCS SP5-2 confocal laser-scanning microscope (Leica Microsystems, Mannheim, Germany) with a Plan Fl20x/0.70 NA objective for the MAP2 stain. Stacks of eight-bit grayscale images were obtained with an axial distance of 3 µm between optical sections each averaged from four successive scans. Finer details (Figure 3B) were taken with a Plan 63x/NA1.32 oil immersion objective. For each optical section the images of one or two fluorochromes were collected sequentially. RGB stacks, montages of RGB optical sections, and maximum-intensity projections were assembled into tables by using ImageJ 1.37k plugins (NIH, USA) and Photoshop (CS6, Adobe Systems, San Jose, CA, USA). Figure images were arranged using CorelDRAW X6 (Corel Corporation, Ottawa, ON, Canada).

### Quantification and analysis

#### Counting of cholinergic neurons

Cholinergic efferent neurons were counted using ChAT immunostaining in the ChAT, MAP2 and CSPG triple-labeled sections (refer to Figure 2). Outlines of the brainstem nuclei were determined in MAP2 and CSPG overview stains. The OC cell counts in the different SOC nuclei were related to a “standard series” of sections spaced 160 µm through the SOC, which was chosen from a young adult experimental animal (refer to Figures 1, 2). The neuron group of vestibular efferents and the neurons of the motor trigeminal nucleus were easy to delineate as most of the neurons in these structures are cholinergic.

**Figure 4:**
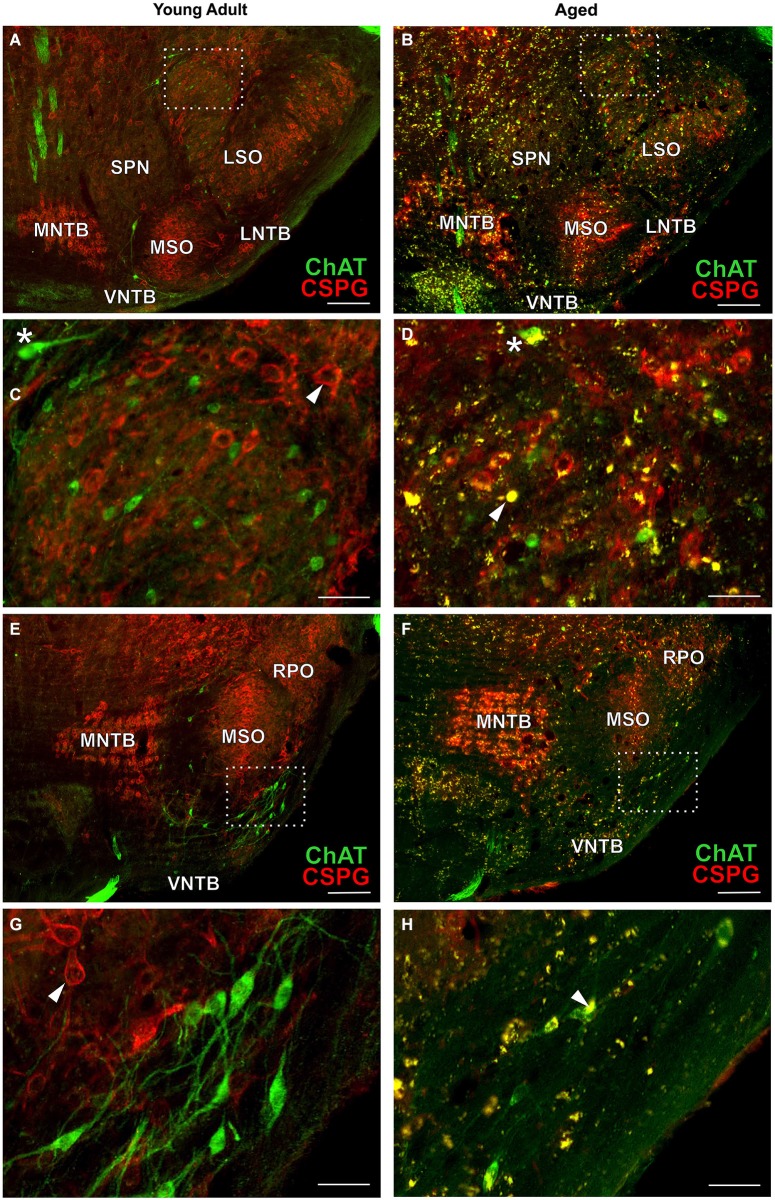
**OC neurons in SOC of young adult and aged gerbils**. ChAT (Alexa Fluor 488, green) and CSPG (Cy 3, red) immunolabeling is shown. Arrowheads point towards a CSPG-positive perineuronal net in **(C,G)**. Note that OC neurons are not ensheathed in perineuronal nets. Autofluorescent lipofuscin granules appear yellow (arrowhead in **D**,**H**). LOC neurons: section at middle of SOC (rc-level 5) in young adult and aged gerbil **(A,B)** with enlargement of rectangular areas in medial LSO in **(C,D)**. MOC neurons: section at rostral SOC (rc-level 9) in young adult and aged gerbil **(E,F)**. VNTB/LNTB area within rectangle in **(E,F)** is enlarged in **(G,H)**. Stars in **(C,D)** mark presumed MOC neurons in DPO/DLPO. The presumed MOC soma in **(D)** is half-filled with lipofuscin granules (yellow). Scale bar in **(A,B,E,F)**: 200 µm; in **(C,D,G,H)**: 50 µm.

In aged animals the presence of autofluorescent lipofuscin granules affects the fluorescent image to some extent (see Figures 4B,D,F,H, 7B,D,F). The lipofuscin granules are excitable by many wavelengths yielding different colors of emission, which makes them distinguishable from secondary fluorescent antibodies that are sensitive only to their specific excitation wavelength. Lipofuscin granules were excited by the excitation wavelength of Alexa Fluor 488 (secondary antibody used to label ChAT in green) as well as by the excitation wavelength of Cy3 (secondary antibody used to label the CSPG-positive perineuronal nets in red) as depicted in Figures 4D,H. Therefore lipofuscin granules appear bright yellow and are clearly distinguished from the green ChAT positive neurons (see Figures 4B,D,F,H, 7B,D,F). Lipofuscin granules are also excitable with the DAPI excitation wavelength (350–360 nm) as shown in Figures 3A,B. Here neurons are stained with anti-MAP2 labeled with Cy3.

**Figure 5:**
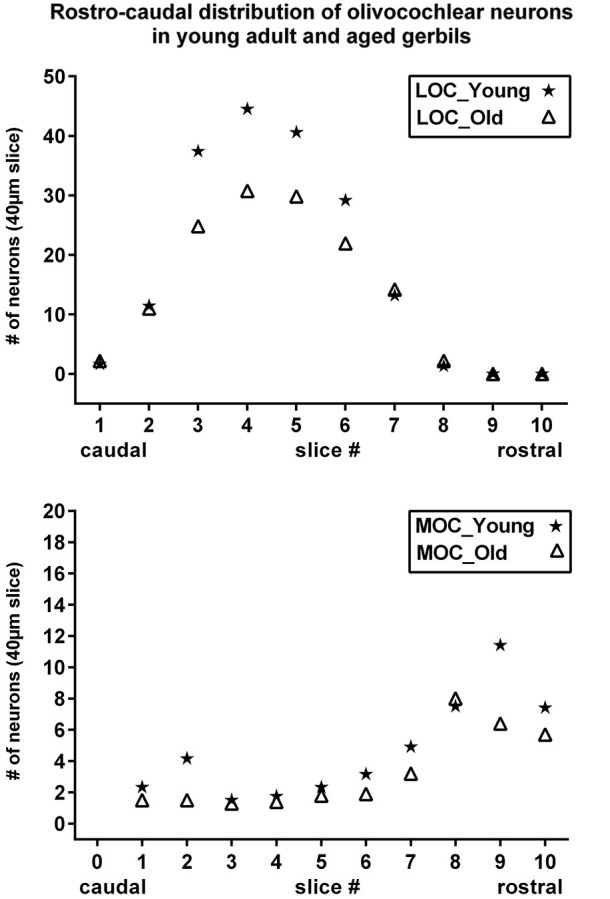
**Rostrocaudal distribution of olivocochlear neurons in young adult and aged gerbils**. Number of LOC (upper) and MOC (lower) neurons are shown as a function of rostrocaudal location in the SOC. The slice numbers (abscissa) refer to the slices in Figure 1, and the values for young adult and aged animals are represented as stars and triangles, respectively. Values give the mean number of neurons in the respective slice of 40 µm thickness as counted in all five animals of each category.

**Figure 6:**
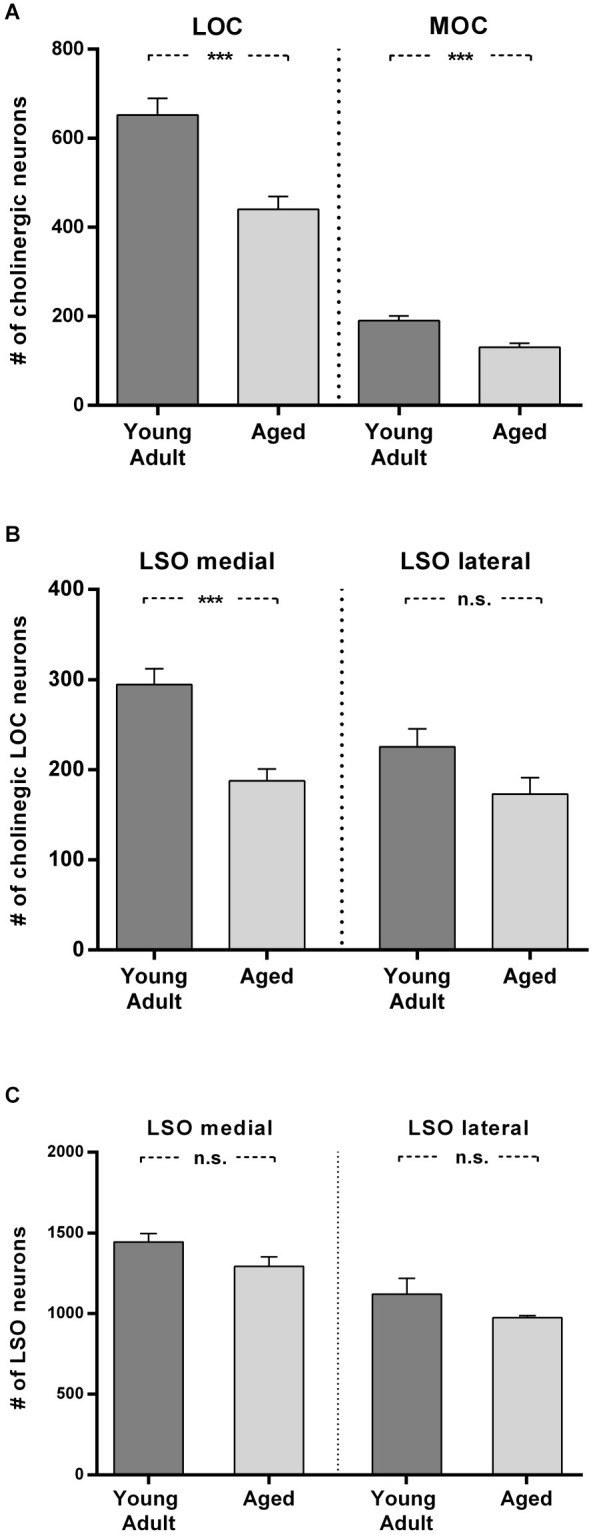
**Loss of olivocochlear (A,B) and LSO neurons in general (C) in aged compared to young adult animals. (A)** The mean number of LOC and MOC neurons in one hemisphere is represented for young adult and aged animals. The decrease in OC numbers in aged animals relative to young adult animals is significant in LOC and MOC neurons (LOC: 24%: *t* = 4.436; df = 18; *p* < 0.001); MOC: 31%, *t* = 4.357; df = 18; *p* < 0.001). **(B)** LOC neuron numbers in medial and lateral part of LSO in young adult and aged animals. The aged vs. young adult decrease of LOC neurons is −36% in the medial LSO portion (*t* = 4.888; df = 18; *p* < 0.001) and −23.4% in the lateral portion (*t* = 1.96; df = 18; n.s.). The difference of LOC numbers in the medial LSO vs. lateral LSO is not significant neither in young adult (*t* = 2,608; df = 18; *p* = 0.0178, n.s.) nor in aged (*t* = 0.657, df = 18; n.s.) animals. **(C)** Total number of LSO neurons within an area of interest of the medial and lateral portion of the LSO for young adult and aged animals. The left pair of columns gives the counts in the medial, the right one the counts in the lateral portion of LSO. LSO neurons exhibit only a moderate loss in both subdivisions with 10% in the medial LSO (*t* = 1.891; df = 6; n.s.) and 13% in the lateral LSO (*t* = 1.471; df = 6; n.s.). Neuron numbers are given as mean ± SEM. Dark gray columns: young adult animals, light gray columns: aged animals. ***p < 0.001.

**Figure 7:**
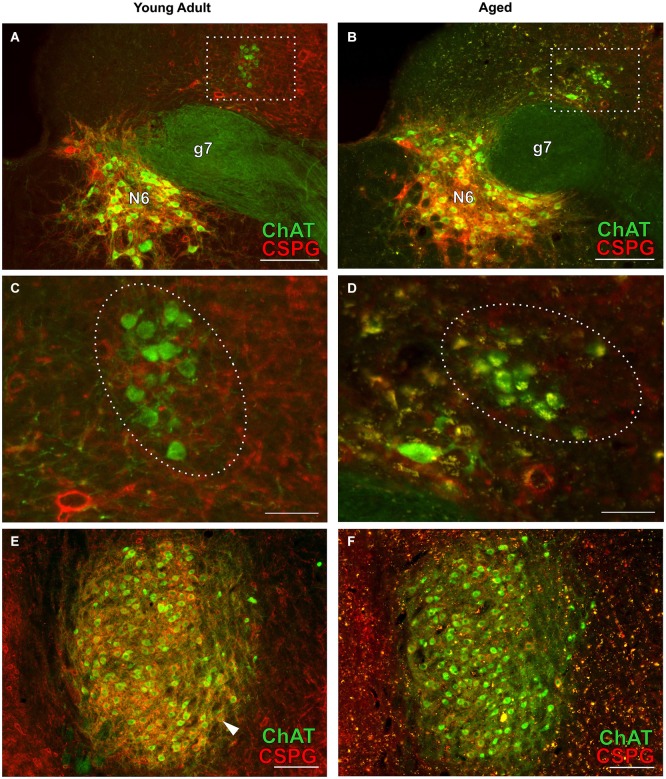
**Cholinergic neurons of the efferent vestibular system (A–D) and of the motor trigeminal nucleus (E,F) in young adult and aged gerbils**. Micrographs were taken from the same series of a pair of young adult and aged gerbils as for the OC examples in Figure 4. ChAT (Alexa Fluor 488, green) and CSPG (Cy 3, red) immunolabeling is shown. The group of vestibular efferents in young adult and aged gerbil is accentuated by rectangles in **(A,B)**. **(C,D)** show enlargements of the rectangles in **(A,B)**, respectively. The groups of ChAT-positive and CSPG-negative efferent vestibular neurons are encircled. **(E,F)** give an overview of the motor trigeminal nucleus. ChAT-positive neurons of the young adult gerbil **(E)** are ensheathed in CSPG-positive perineuronal nets (arrowhead in **E**) whereas most cholinergic neurons of the aged gerbil **(F)** show degraded perineuronal nets and a comparably low amount of lipofuscin granules (yellow). N6: abducens nucleus; g7: genu of the facial nerve. Scale bar in **(A,B,E,F)**: 200 µm; in **(C,D)**: 50 µm.

Based on the assumption that a soma that is not completely filled with lipofuscin granules is functional, we conservatively counted in aged animals all somata in which immunoreactivity for ChAT was still detectable.

The individual cholinergic somata were clearly identifiable within 40 µm slices. Double counting of somata was excluded as neurons were counted in every fourth slide in the series, i.e., at a distance of 160 µm between evaluated slices. The maximum diameter of cholinergic OC, vestibular and trigeminal somata are all below 50 µm and thus far below this distance (160 µm), so that double-counting of parts of a soma split due to slicing could not occur in the slices used for counting (120 µm apart). Cholinergic neurons in the images were marked on a separate layer in Photoshop (CS6 Extended, Adobe Systems, San Jose, CA, USA) and then counted with the aid of ImageJ plug-in “cell counter” (Kurt De Vos, Univ Sheffield, Academic Neurology^1^), separately on both sides of the brain. The cell numbers in the three interspersed slices between two evaluated slices were estimated by interpolation (Gleich et al., 2004; Kaiser et al., 2011). The underlying assumption was that the number of olivocochlear neurons does not change abruptly within the 160 µm of rostrocaudal extension and develops monotonically (Sanes et al., 1989). The counted and interpolated neuron numbers together represent the total number of cholinergic OC neurons within a virtual slice of 160 µm rostrocaudal width. Numbers of cholinergic neurons are always given for one side of the brain throughout the report.

#### Counting of LSO neurons

To assess the age-related loss of neurons in the SOC in general, MAP2 stained somata were counted in four pairs of young adult and aged animals. Somata were counted within restricted areas of interest (277 µm × 277 µm) of the lateral and medial limb of the LSO, respectively, in four sections spaced 160 µm corresponding to the rostrocaudal levels 3–6 of the standard reference series (Figure 1). Due to the much higher density of neurons (compared to cholinergic neurons) counting had to be performed on optical slices obtained from the 40 µm thick sections with the confocal microscope. Thirteen consecutive optical sections of 3 µm thickness each were used. The soma of each neuron was tracked individually through the stack and counted once in order to avoid double counting (West, 1993).

### Statistical analysis

Counting results of individuals and counts within or between groups were statistically treated with the program Prism6 (for Windows, GraphPad Software, San Diego, CA, USA^2^). Comparisons were performed with an unpaired *t*-test. Statistical significance is indicated with the *t* value, the degrees of freedom (df) and the *p* value. These indications are also included in the figure legends. Statistical significance was determined applying a criterion of *P* < 0.01.

## Results

### OC neurons

The distribution of cholinergic neurons comprising LOC and MOC neurons in the SOC was evaluated in five pairs of healthy young adult gerbils (3–5 months old) and aged gerbils (between 2.5 and 3.5 years).

The distribution in young adult gerbils is in general similar to what has already been described in rodents (see Section Introduction), and is represented schematically in 10 standard slices through the SOC in Figure 1. The number of cholinergic neurons as a function of rostrocaudal location within the SOC is compiled in Figure 5 for LOC and MOC neurons in parallel for young adult and aged animals, respectively. The small LOC neurons were numerous within the LSO but distributed sparsely in the border region to periolivary nuclei, notably in the dorsolateral periolivary nucleus (DLPO). A few small cholinergic neurons were found in untypical locations in the ventral and lateral nuclei of the trapezoid body (VNTB/LNTB Figure 1, rc-levels 3–10), in the dorsal/dorsolateral periolivary nuclei rostral to LSO and in the region of the anterolateral and rostral periolivary nuclei (DPO/DLPO, ALPO/RPO, Figure 1, rc-level 8–10). These neurons were analyzed separately. They displayed large individual variation but showed no significant difference between young adult and aged animals. MOC neurons occurred mostly in VNTB and in the ventromedial periolivary nucleus (VMPO). Some large cholinergic neurons were also found in the dorsal and dorsolateral periolivary nuclei (DPO/DLPO; Figure 1, rc-level 3–10; and Figures 4A–D). These neurons were presumed to be MOC neurons as they showed an age-related decline like the main group of MOC neurons (see below). No cholinergic neurons were detected in the medial superior olive (MSO), the medial nucleus of the trapezoid body (MNTB) and almost none in the superior periolivary nucleus (SPN).

### Comparison of OC neurons in young adult and aged animals

#### Immunolabeling

Typical examples of OC neurons from a pair of young adult and aged gerbils are depicted in the upper panels of Figure 4 for LOC (Figures 4A–D, rc-level 5) and in the lower panels for MOC (Figures 4E–H, rc-level 9). The principal nuclei of the SOC were prominently marked by the presence of perineuronal nets around their principal neurons as seen by the positive CSPG stain (red). In comparison, SPN, VMPO and VNTB staining of the nets was weak (Figures 4A,B,E,F; for detailed delineation of nuclei refer to Figure 1). LOC and MOC neurons were immunopositive for ChAT (green) but CSPG-negative, i.e., they did not possess perineuronal nets (Figures 4C,D,G,H, respectively). Overall we observed a key qualitative change of OC neurons in the aged brains: sections of aged animals were characterized by auto-fluorescent lipofuscin granules that accumulated in the neuronal somata during aging. As the fluorescence of lipofuscin granules is excited by a broad spectrum of wavelength, the granules stood out yellowish against the green cholinergic structures and the red perineuronal nets (see Figure 4, enlargements D and H). Perineuronal nets tended to fade, clump and dissolve with old age, which gave them a blurry appearance, but still helped to unequivocally delineate the SOC nuclei (Figures 4B,F). We also observed some of the spongiform lesions (Figures 4F,H) typical of aged gerbil brainstem tissue (Ostapoff and Morest, 1989).

#### Cell counts

Maximum cell counts of LOC neurons were found at rc-level 4 and 5, where LSO was at its largest cross section (Figures 1, 5, upper panel, stars). Loss of LOC neurons in aged animals was prominent between rc-levels 3 and 6 (Figure 5, upper panel, triangles) which comprised the major volume of the LSO. MOC neurons extended over the entire rostrocaudal range of the SOC. Their number increased rostrally with a maximum between rc-levels 7 and 10 (Figure 5, lower panel, stars). In aged animals the loss of MOC cells spread throughout the rostrocaudal range with a maximum loss at rc-level 9 (Figure 5, lower panel, triangles). The counts of cholinergic OC neurons in young adult animals were fairly constant across individuals. Mean total number and standard deviation of mean (SEM) of LOC neurons per hemisphere and animal was 724 ± 43 (*N* = 10). MOC neurons amounted to 190 ± 10 neurons per hemisphere (*N* = 10). LOC neurons outnumbered MOC neurons almost by a factor of four (with LOC comprising 79% vs. MOC comprising 21%, of all cholinergic OC neurons).

The mean total numbers in aged animals were 551 ± 26 (*N* = 10) for LOC and 131 ± 9 (*N* = 10) for MOC neurons. The LOC to MOC ratio had virtually not changed in aged animals (80.9% vs. 19.1%, respectively). The average OC neuron counts of left and right hemispheres yielded similar numbers, and there was no sign of lateralization.

In Figure 6A the means of total LOC and total MOC numbers for the young adult and aged cohort are contrasted and deviations are given as standard error of means (SEM). The loss of LOC neurons in the aged animals compared to young adult individuals of 24% was significant (*t* = 4.436; df = 18; *p* < 0.001). Aged animals also showed a significant loss of MOC neurons of 31% (*t* = 4.357; df = 18; *p* < 0.001). LOC neuron number in the LSO turned out to be not equal (Figure 6B), with significantly more LOC neurons in the medial limb than in the lateral limb of LSO in young adult animals (+23.4%; *t* = 2.608; df = 18; *p* = 0.018). The loss of LOC neurons in aged animals relative to young adult animals was significant in the medial part of LSO (−36%; *t* = 4.888; df = 18; *p* < 0.001). There was a trend of mean LOC decrease in the lateral LSO portion in aged animals which, however, was statistically not significant (23.4%; *t* = 1.96; df = 18; *p* < 0.1, n.s.). LOC neuron number in the medial and lateral limb of the LSO of aged animals was therefore less different (7.8%; *t* = 0.657; df = 18; *p* = 0.5195, n.s.).

To find out whether a general decrease in neuron numbers with age could also explain the differential loss of LOC neurons, the total number of LSO neurons was counted in a standard area of interest within medial and lateral part of LSO in young adult and aged animals. Figure 6C shows that the age-related loss of LSO neurons was virtually equal in the medial and lateral LSO limb and amounted to 10% and 13%, respectively (medial: 10%; *t* = 1.891; df = 6; n.s.; and lateral: −13%; *t* = 1.471; df = 6; n.s.). The average loss in LSO neurons of only about 12% cannot account for the loss in OC neurons, which was almost three times as high. The larger loss of LOC neurons in the medial compared to lateral subdivision of LSO did also not follow the pattern of general LSO neuron number decrease with age in the respective LSO limbs.

### Comparison of age-related neuronal loss of auditory with vestibular and motor trigeminal efferents

**Figure 8:**
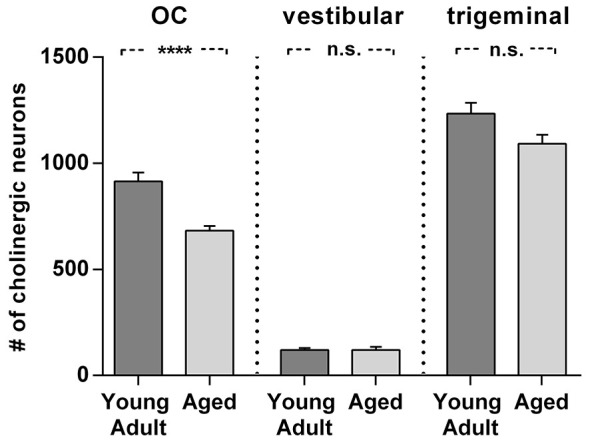
**Comparison of age related neuron loss in three cholinergic efferent systems**. There is considerable loss in the olivocochlear efferent system (left) of 26% (*t* = 4,984; df = 18; *p* < 0.0001), whereas the vestibular efferent system (middle) is not affected by age and the trigeminal motor system (right) shows only a moderate neuronal loss of 12% (*t* = 2.155; df = 18; n.s.). Neuron numbers are given as mean ± SEM. Dark gray columns: young adult animals, light gray columns: aged animals. **** *p* < 0.0001.

Cholinergic vestibular efferent neurons from young adult and aged gerbils (same animals as in Figure 4) are depicted in the upper two panels of Figure 7. Like OC neurons, the medium sized round shaped cholinergic vestibular efferent neurons stained positive for ChAT, but were not ensheathed by CSPG-positive neuronal nets. Compared to OC neurons in the aged animal (Figures 4D,H) cholinergic vestibular efferents were only moderately loaded with lipofuscin granules (Figure 7D). Their number was not significantly reduced as shown in Figure 8 (young adult: 119 ± 11, *n* = 10; aged: 120 ± 14, *n* = 10; *t* = 0.0449; df = 18; n.s.).

The large efferent motor trigeminal neurons (Figures 7E,F) stained positive for ChAT, and also for CSPG. The salient CSPG-positive perineuronal nets distinguished them from the auditory and cholinergic vestibular efferents. In aged animals these nets around the somata showed marked deterioration (Figure 7F). The neurons accumulated only moderate amounts of lipofuscin granules and their number was slightly reduced, but this loss was barely significant (see Figure 8, young adult: 1234 ± 51, *n* = 10; aged: 1092 ± 66, *n* = 10; *t* = 2.155; df = 18; n.s.).

## Discussion

The present study quantified the age-related loss of cholinergic efferent neurons in the auditory and vestibular systems and motor trigeminal nucleus. It revealed a loss of almost one third of the auditory efferents in aged gerbils in contrast to no or almost no neuronal loss of cholinergic vestibular efferents and neurons of the motor trigeminal nucleus. These observations suggest differential effects of aging on these three cholinergic brainstem systems.

The number and ratio of LOC and MOC neurons in young adult gerbils are in accord with the findings of Aschoff et al. (1988). The remarkable decline of auditory efferents with age was 31% for MOC and 24% for LOC neurons. Hence, the ratio of MOC to LOC neurons remained virtually constant in aged animals. The decline was equal in both hemispheres and showed little inter-individual variability. The decline of LOC neurons within LSO was significantly larger in the medial LSO than in its lateral part. This could not be explained by the comparably moderate and equally distributed general cell loss within LSO in aged animals of roughly 12%. Also, Gleich et al. (2004) observed that the number of neurons within the gerbil’s LSO is only marginally affected by age (for review on age related changes in the SOC see Caspary et al., 2008). As auditory efferent neurons exert central feedback control on the cochlea and modulate information processing, their high loss should have serious functional consequences and could contribute to age-related hearing deficits.

MOC neurons protect hair cells from acoustic injury via a sound-evoked efferent reflex and are known to be involved in age-related decline of auditory processing (e.g., Kim et al., 2002; Jacobson et al., 2003; Fu et al., 2010). The functional role of the LOC neurons is less clear. LOCs could potentially prevent glutamate excitotoxicity at synaptic terminals of the auditory nerve (Pujol and Puel, 1999) by modulating the excitability of the nerve fibers (Ruel et al., 2001; Groff and Liberman, 2003). Recently, participation of olivo-cochlear neurons, especially MOCs in “hidden hearing loss” (Schaette and McAlpine, 2011) has been demonstrated (Furman et al., 2013): OC neurons have a protective effect against the selective loss of high-threshold auditory nerve fibers induced by moderate noise exposure (Maison et al., 2013). These fibers are most important for hearing-in-noise, which becomes increasingly more difficult with age, and is not necessarily correlated to a rise in auditory thresholds (Liberman et al., 2014). Thus age-related impairment of hearing-in-noise could result from the loss of efferent terminals.

Within the LSO a lateromedial tonotopic gradient from low to high frequencies is well established (e.g., gerbil: Sanes et al., 1989; rat: Kelly et al., 1998; cat: Tsuchitani and Bodreau, 1966; dog: Goldberg and Brown, 1968), and a lateral and medial limb of LSO can also be distinguished neuroanatomically. Consistent with previous observations of Kaiser et al. (2011), we found a higher density of LOC neurons in the medial, high-frequency processing LSO limb in young animals compared to the lateral low-frequency portion and significant age related loss of LOC neurons was only found in this portion of the LSO. Liberman et al. (2014) showed that MOC effects are most important to efferent-mediated protection in the apical half of the cochlea, whereas LOC contributions dominate in the basal half of the cochlear partition where high frequencies are processed. Thus the significant age-related loss of LOC neurons found in the medial portion of the LSO would predominantly affect the high frequency processing capabilities of the animals. This is also consistent with observations of age-related hearing loss in the high frequency range in gerbils and humans (e.g., Mills et al., 1990).

The cholinergic efferent vestibular feedback pathway, however, is not subject to such neuronal loss in old animals despite sharing a lot of features with the auditory efferent system. Efferent fiber stimulation results in complex effects on the activity of vestibular afferent neurons by increasing, inhibiting or having mixed biphasic effects on the electrical discharge of the afferent neurons (Soto and Vega, 2010). The functional significance of these effects is, however, still not fully understood.

Differences in functional shortcomings in the auditory and the vestibular system with age suggest a prevalence of loss of auditory efferents. In a longitudinal study Enrietto et al. (1999) investigated age-related decrease in auditory (pure tone and speech perception, detection threshold and speech discrimination score tested psychophysically) and vestibular responses (VOR measurements) in healthy older human subjects. The faster decrease in auditory responses was not correlated with age-related changes in the vestibular system. They concluded that the two systems may age at different rates in the same individual. Hence age-related dysfunction of the auditory system need not be correlated with any deterioration of the vestibular system, as has also been shown in C57BL/6 mice (Shiga et al., 2005). In contrast to the auditory system, age-associated degeneration of the peripheral vestibular system in C57BL/6 mice was not significantly correlated with any age-dependent changes in function and followed a different time course when compared to changes in auditory function.

The age-related loss of cholinergic efferent neurons of the motor trigeminal nucleus was found to be minimal in gerbils in accord with an earlier study of Sturrock (1987) that showed only sparse neuronal loss of trigeminal neurons in the aged mouse. However, other than auditory and cholinergic vestibular efferent neurons, motor trigeminal neurons do possess perineuronal nets and aging dramatically affects these nets. Among several other functions attributed to perineuronal nets, they have been shown to protect neurons from oxidative damage (Suttkus et al., 2012, 2014), but are themselves sensitive to excess oxidative stress (Cabungcal et al., 2013). Perineuronal nets might therefore be an effective protection from early functional impairment of trigeminal neurons with age. However, arguing against this, vestibular efferent neurons in the gerbil do not possess perineuronal nets and also showed no neuronal loss with aging.

Our results are in line with recent findings showing that during normal aging in humans and in animals, there is only a modest decline of neuronal number (probably no more than 10% (Morrison and Hof, 1997)). However, there is a specific age-related neuron loss in restricted neuronal populations of the nervous system (Pannese, 2011). In addition to a loss of cholinergic neurons, a functional decline with age might also be caused by dendritic, synaptic, and axonal degeneration as well as a decrease in trophic conditions of these neurons (Schliebs and Arendt, 2011).

Summarizing our findings, there is a significant loss in the auditory efferent system in senescent gerbils, with no counterpart in the vestibular efferent system or in the neuron population of the motor trigeminal nucleus. The observations support the concept of different mechanisms for age-associated changes in the different cholinergic systems of the brain stem.

## Author contributions

Susanne Radtke-Schuller: conceived project, conducted experiments, analyzed and interpreted data, wrote manuscript. Sabine Seeler: conducted immunostaining, performed cell counts. Benedikt Grothe: conceived project jointly with Susanne Radtke-Schuller, interpreted data and jointly wrote manuscript.

## Conflict of interest statement

The authors declare that the research was conducted in the absence of any commercial or financial relationships that could be construed as a potential conflict of interest.

## Footnotes

^1^http://rsb.info.nih.gov/ij/plugins/cell-counter.html

^2^www.graphpad.com
